# Maintaining Warm, Trusting Relationships with Brands: Increased Temperature Perceptions after Thinking of Communal Brands

**DOI:** 10.1371/journal.pone.0125194

**Published:** 2015-04-27

**Authors:** Hans IJzerman, Janneke A. Janssen, James A. Coan

**Affiliations:** 1 VU University, Department of Clinical Psychology, Amsterdam, the Netherlands; 2 Tilburg University, Department of Social Psychology, School of Social & Behavioral Sciences, Tilburg, the Netherlands; 3 University of Virginia, Department of Psychology, Charlottesville, Virginia, United States of America; Universiteit Utrecht, NETHERLANDS

## Abstract

Classical theories on interpersonal relations have long suggested that social interactions are influenced by sensation, such as the experience of warmth. Past empirical work now confirms that perceived differences in temperature impact how people form thoughts about relationships. The present work first integrates our knowledge database on brand research with this idea of “grounded social cognition”. It then leverages a large sample (total *N* = 2,552) toward elucidating links between estimates of temperature and positive versus negative evaluations of communal brands. In five studies, the authors have found that thinking about positively (vs. negatively) perceived communal brands leads to heightened temperature estimates. A meta-analysis of the five studies shows a small but consistent effect in this noisy environment, *r* = .11, 95% CI, .05, .18. Exploratory analyses in Studies 1a and b further suggest that temperature perceptions mediate the (significant) relationship between perceived communality and willingness to purchase from the brand. The authors discuss implications for theory and practice and consider the effects from a Social Baseline Perspective.

## Introduction

One of the best-known advertisements for Coca-Cola features large, illuminated trucks winding their way through a winter landscape, offering relief from winter’s coldness in the form of warmth (and light). Amongst other things, Coca-Cola’s marketers are likely seeking to form trusting, warm relationships with their consumers. It may gratify these marketers to learn that real physically warm feelings may be instilled in consumers when dealing with brands that customers perceive to be trustworthy. As it stands, findings in the cognitive sciences suggest that close relationships in general instill an increased sense of physical warmth, and here, in several high-powered studies, we specify this to people’s perceived relationships with brands.

We find that when offering people brands they rate as more trustworthy, they estimate ambient temperature to be higher (Studies 1a&b). In addition, when participants themselves pick a trustworthy (vs. untrustworthy) brand they also perceive an elevated ambient temperature (Studies 2a&b). We ran high-powered studies, and provide greater confidence in the research hypotheses than has previously been done. Finally, a novel—and unexpected finding—is that temperature estimates are, but subjective feelings of warmth are not, reliably influenced by communality perceptions of brands. This study further refines the model of this type of work, as large samples have become a dire necessity in psychological research [1]. Together, these findings offer us insights—both novel and solid—into processes underlying the maintenance of our relationships with brands.

## Brand Relationships

Because brands in fact fulfill relationship functions, in ways that typically seems reserved for our relationships with people. Work on collectors of Hallmark and Coca-Cola products has even revealed that people maintain relationships with brands, suggesting “to these collectors, Hallmark is like a lover; dating, seducing, and tying the knot on an enduring and dependent relationship” [2]. Maintaining relationships with brands extends to non-collectors, as people maintain affectionate feelings for products not unlike people’s social attachments. Ahuvia [3] interviewed 69 participants, of which only 2 indicated love to being reserved strictly for human beings—and not for objects or products. When the researcher asked participants whether love towards consumer products could be described as being “true” and “real”, still 72% of the respondents claimed it being so. Theoretically, Shimp and Madden [4] designed a model of “consumer-object love”, capturing components traditionally reserved to love between people [5]. On an even more anecdotal note, Richins [6] identified a set of consumption emotion descriptors to describe emotional attachments to brands. One of their established descriptors, love, is measured by the degree of *warm-heartedness* towards a product.

## Brand Personality

In line with the idea that brands serve relationship functions, people utilize person perception judgments to judge their brands. People may for example perceive a brand as cool, innovative, and athletic or even authentic, practical, stylish, and trustworthy. This application of human personality traits has been referred to as “brand personality”, defined as "the set of human characteristics associated with a brand" [7]. Other people may be perceived as an individual (agency) or as a part of something bigger, involving relationships (communion) [8]. Over the years, researchers have established communion and agency as the fundamental dimensions underlying social perception [9; 10]. Communion, also known as (psychological) warmth, is the most dominant, and consists of personality traits allowing people to identify others as trustworthy (such as helpfulness, honesty, and cooperation). The second dimension, agency, consists of traits that are often used to identify whether someone is competent, such as efficiency, persistence, and energy [11]. The concept of agency specifically concerns traits perceived to relate to self-interest and the achievement of personal goals [12; 13]. And within the consumer science literature, it is now well established that people project such personality traits onto brands [14; 15].

## Sensory Experiences of Warmth as Building Block for Social Relationships

Named dimensions regarding personality and social interaction have by now been well established (for a recent review, see [16]). But in more recent years, researchers have supplemented the well-known literature on mentally representing relationships and people with *grounded* cognitive processes. After the cognitive revolution, the dominant metaphor for the human mind became that of a computer, with mental content driven by abstract, amodal representations, independent from their original experiential bases. This presented researchers with what has become known as “the symbol grounding problem” [17; 18].

Broadly speaking, there are two pathways that are important to understand relationship experiences from this grounded cognition perspective. The first is an evolutionary perspective—the degree to which people rely on innate templates for interaction. The second, more relevant perspective relies on learning: Harnad [17] postulated that in the early learning of abstract mental representations, people rely on “iconic representations”, which are “internal analog transforms of the projections of distal objects on [their] sensory surfaces” [19]. Concretely, this means that, at least in Harnad’s view, people learn mental concepts through sensory experiences, like seeing, feeling, touching, or tasting. These mental representations become directly stored in people’s perceptual systems; in the case of retrieval, people simulate the original perceptual state when the original information is re-activated. Exemplary of this theory is that when researchers primed participants with the word “kick”, the associated motor areas in the brain were activated [20]. Another example of this idea is that the verb “to smile” activates the zygomaticus (smiling) muscle to a greater degree than the adjective “a smile” [21]. This is not to say that simulations are exact recordings. Simulations are not unlike any other representational system that is incomplete in its recording, while it is similarly dependent upon goals and social contexts [31]. The idea that cognition is grounded in sensory experiences indeed “reflects the assumption that cognition is typically grounded in multiple ways, including simulations, situated action, and, on occasion, bodily states” (p. 619; [32], see also [33]).

Harnad’s perspective is complemented by Bowlby’s [22] attachment theory. Bowlby postulated that people affiliate early on through their “basic perceptual building bricks” (see also [23]). These perceptual building blocks contribute to what Bowlby coined to be an “internal model” of their social world. In the past decade, researchers have started to relate sensory cues to internal models of close relationships. Specifically, Williams and Bargh [24] revealed that briefly holding a physically warm cup leads to judge others as being more communal (but see [25], for skepticism of this finding), while IJzerman and Semin [26] found that temperature affects people’s social cognitions systemically, as cues of warmth (vs. coldness) induce people to feel closer to a stranger, let them use more relational language, and lets them detect more perceptual relationships (for a high-powered successful replication, see [27]). In other words, when thinking of people’s maintenance of their relationships with brands, temperature perceptions and sensation may well form an important part. The relevance of warm sensations for cognitive models of relationships is further expressed as priming social exclusion and psychological distance leads to participants estimating ambient temperature as lower [28;29] (for an overview, see [30]). These findings can be summarized as that people form “working models” of close relationships that relate feelings of communion with temperature.

## Summary

The present research seeks to extend this literature to people’s maintenance with brands. Particularly relevant for the present work are findings that the estimation of room temperature are also found when reminding people of positive (vs. negative) communal traits of others and self, which leads to higher (lower) of temperature perception [34]. Szymkow, Chandler, IJzerman, Parchukowski and Wojciszke’s effect was driven by the concept of communion, and not by positive or negative valence, as the effects were not detected for positive (vs. negative) agency. Given that people conceptualize relationships to brands in much similar ways to relationships with human beings, we hypothesized that thinking of positive communal brands evoke heightened temperature perceptions in very similar ways as it does in response to trustworthy humans. This is important for theory, as it can clearly inform us how people form and maintain relationships with brands, and for practice, as temperature manipulations or measurement can inform practitioners on how to form relationships with their consumers.

## Overview of Studies

In the present research, we examined whether thinking about communal brands led to increased temperature perceptions. First, we tested whether people who are exposed to specific positively (vs. negatively) valenced communal brands afterwards provide higher estimates of ambient temperature (Studies 1a&b), and investigate whether these effects are independent of valence. Second, we tested whether priming participants with positively (vs. negatively) valenced communal traits of a brand of their own choosing leads to higher temperature perceptions (Studies 2a&b&c). On top of that, we also explored whether the effects of communality that we have typically found extend from temperature perception to subjective warmth. We did not have a clear expectation regarding this variable beforehand, but found it not to be influenced.

The overarching goal was to investigate whether combining communal traits with brands leads to estimating ambient temperature as higher. Beyond that our research extends this area of investigation to relationships with brands, we offer greater confidence in these findings by investigating large samples, while being one of the first ones to extend this area of research to non-student samples [35] (for an exception, see [36]). For all studies, we report how we determined our sample size, all data exclusions, all manipulations, and all measures in the study [37]. All data and analyses are available on the Open Science Framework’s website [38]. Any exploratory analyses on additional variables are reported on the project’s page on the Open Science Framework.

## Studies 1a&b

In the first two studies we investigated whether exposing people with communal brands leads to higher temperature estimations. We report them together here because of their high degree of similarity. In a first test, we related communality to temperature by 1) priming participants with brands that were rated in a pilot test as positively communal (Nintendo) and negatively communal (Walmart) and 2) by measuring communality of these brands by the present group of participants, and relating them to temperature perceptions. Initially, we had hypothesized to find a condition effect, but after detecting a relationship between communality and temperature perception, we updated our hypothesis in Study 1b. In a second test, we thus repeated Study 1a, and also expanded our list of brands (Walmart, Nintendo, Google, Sony, & Coca-Cola). We hypothesized that in both cases we would find a relationship between temperature and the communality of the brand.

This research was conducted in accordance with ethical standards and publications set forth by the American Psychological Association. For all studies we did not seek approval from the ethics committee since only adults were involved in the study and there was no risk for either emotional or physical health. The authors did not physically interact with the participants and obtained consent prior to running the study via approval on the screen. Beforehand, we did not inform participants about the full purpose of the study, but they were fully debriefed afterwards. Based on the data we downloaded from Qualtrics, we are not able to identify our participants. In all studies, participants were paid $0.40 in exchange for their participation. Finally, all participants completed our tasks anonymously and we did not collect health related questions.

### Participants and design

For Study 1a, based on an a priori power analysis, with a Cohen’s *f* of. 20, power of. 95, and an alpha of. 05 [39], we recruited 328 MTurk workers. Before conducting any analyses, we removed 3 workers because they did not know the brand of the condition they were in, and 13 more because they looked at their thermostat prior to estimating temperature. For Study 1a, this left us with a final sample of 312 workers (*M*_age_ = 31.38, *SD*_age_ = 10.57; 37.5% female). For Study 1b, we updated our power analysis based on the detected effect size for Nintendo (*r* =. 24), which would give us a sample size of 215. In order to have enough participants, we again aimed for 312 MTurk workers (but ended up with 318). We excluded 1 participant for not knowing the brand and 23 for admitting to look at the thermostat before estimating temperature. For Study 1b, this left us with a total of 294 Workers (*M*_age_ = 27.61, *SD*_age_ = 8.49; 27.9% female).

### Procedure and materials

Participants entered an online questionnaire in Qualtrics via a link at Amazon Mechanical Turk’s website. In Study 1a, participants were randomly assigned to a positive communal brand prime (Nintendo; *N* = 159) or negative communal brand prime (Walmart; *N* = 153) condition. In Study 1b, participants were randomly assigned to one of five brands (*N*_Nintendo_ = 57; *N*_Walmart_ = 59; *N*_CocaCola_ = 64; *N*_Sony_ = 58; *N*_Google_ = 56).

First, participants took part in a categorization task, during which six different brand images of the condition brand were presented. They were instructed to divide the logos into two categories by dragging and dropping the logos on the computer screen in the box named “most preferred images” or “least preferred images”. Next, under the guise of an inquiry of participants’ physical circumstances, they were asked to indicate their estimate of ambient temperature in their own environment on a 20-point slider scale (60°F—80°F), which also had the option to indicate “not applicable” when their temperature estimate was out of this range (1a *N* = 1; 1b *N* = 0).

The instruction further stressed that participants were not allowed to look at their actual thermostat and—as another failsafe—we also asked participants afterwards whether they checked their thermostat before estimating ambient temperature. Furthermore, participants rated whether they thought brands were fitting with 20 personality traits that were rated on positive communality, negative communality, positive agency, and negative agency, from our first pretest on a 7-point scale (1 = *strongly disagree*, 7 = *strongly agree;* Study 1a Cronbach’s **α** COM_pos_ =. 92; COM_neg_ =. 87; AGE_pos_ =. 78; AGE_neg_ =. 79; Study 1b **α** COM_pos_ =. 84; COM_neg_ =. 78; AGE_pos_ =. 73; AGE_neg_ =. 77). We reversed both negative scales and averaged them with our positive scales into an Averaged Communality and Averaged Agency score.

### Results

Contrary to what we expected, an independent sample *t*-test did not reveal a main effect of condition (Nintendo vs. Walmart) on temperature estimates for Study 1a (*t* < 1). However, examining bivariate correlations, Study 1a revealed a positive significant correlation between Averaged Communality and the temperature estimate, *r* =. 11, *p* =. 044 (but a negative correlation with subjective warmth, *r* = -.18, *p* <. 01). The detected effect was not due to valence, as Averaged Agency did not correlate significantly with temperature estimates, *r* =. 06, *p* =. 28.

We also checked for the two brands separately, and saw that the relationship between communality and temperature estimates was entirely driven by Nintendo (*r* =. 24, *p* =. 002), and not Walmart (*r* =. 09, *p* =. 25), which caused us to run a second study, with an increased number of brands included based on our updated hypothesis. In Study 1b, a univariate analysis of variance, including the dummy coded brands Nintendo, Walmart, Coca-Cola, Sony, and Google, revealed a significant positive relationship between Averaged Communality and temperature estimates, η_p_^2^ =. 019, *F*(1, 293) = 5.65, *p* <. 001, with again no effect for Averaged Agency (*F* < 1), but the effect for Walmart was again weaker. There were no significant interactions between the dummy coded variables and communality for all brands, and Walmart was marginally significant (Walmart, *p* =. 06; all other ps >. 21). Also, there was no relationship between communality and subjective feelings of warmth, *F*(1, 307) = 1.72, *p* =. 19. Together, these two studies thus confirm our suggested hypothesis that exposing participants to communal brands is related to heightened temperature perceptions.

### Discussion

In our first two high-powered studies, we found that being exposed to a communal brand was related to higher temperature perceptions. These effects were small (with *r*s =. 10 and η_p_^2^ =. 019), which should not be surprising, as we conducted a study with very low experimental control. In the first study, we thought that a prime with (pre-tested) communal brands would lead to differences in temperature estimates. We did not detect this effect, but instead found a relationship between how communal people found the brand and the temperature estimate. Further, when we (post-hoc) examined variation in the communality ratings of the brands, we saw that there was substantial variation in Study 1a (with our positive communal brand, Nintendo, ranging from 2.60 to 7 on positive communality ratings; *M* = 5.48, *SD* =. 72) and our negative communal brand, Walmart (ranging from 1 to 7 on positive communality ratings; *M* = 3.68, *SD* = 1.31) only being slightly lower than the neutral midpoint of the scale.

In addition, we detected effects on the hypothesized temperature differences, and no consistent effect on subjective warmth. To maximize chances to let participants think of positive and negative communal brands, and to minimize idiosyncratic differences in trust of brands, in the next three studies, we sought to further establish the causal link between communal primes and temperature perceptions by letting participants choose their own positive or negative communal brand. We again used the subjective warmth measure to explore whether our effects extend from temperature perception to subjective warmth.

## Studies 2a&b&c

### Participants and design

Our first power analysis was conducted prior to Study 1a, as Study 2a was conducted approximately at the same time, immediately followed by Study 2c. We thus calculated our sample in G*POWER with a Cohen’s *f* of. 20, power of. 95, and an alpha of. 05 [40] and recruited 326 MTurk workers. We excluded 8 workers who did not follow instructions (naming a brand that they themselves scored as 4 or below in positive communality for the positive communal condition et vice versa). We then excluded an additional 12 workers who checked the thermostat prior to estimating temperature, leaving us with *N* = 306 (*M*_age_ = 31.06, *SD*_age_ = 11.40; 38.2% female). Two additional participants did not provide temperature estimations. Our participants were randomly distributed over our positive communal (*N* = 152) and negative communal (*N* = 151) conditions (2 did not provide temperature estimations). In Study 2b&c, which serve as improved replications of Study 2a, we adjusted our sample size calculation based on our Study 2a results. For Study 2b this gave us a suggested sample size of 591 (we aimed to collect 600 workers). Due to an oversight, we collected an extra 21 workers. Two participants did not fill in the entire questionnaire. We excluded 15 workers due to not following instructions and 47 for looking at the thermostat prior to estimating temperature, leaving us with 557 workers (*M*_age_ = 29.45, *SD*_age_ = 9.29; 31.4% female) in total (all of the remaining participants provided temperature estimates). These were randomly distributed over our positive communal (*N* = 273) and negative communal (*N* = 284) conditions. For Study 2c we recruited 592 MTurk workers of which 14 were excluded for not following instructions and 35 admitted to looking at the thermostat prior estimation of temperature. This resulted in a total of *N* = 543 (*M*_age_ = 31.08 = 3, *SD*_age_ = 10.97; 40.4% female) participants, randomly distributed over the positive communal (*N* = 268) and negative communal (*N* = 276) conditions.

### Procedure and materials

Participants again entered an online questionnaire in Qualtrics via a link at Amazon Mechanical Turk’s website. In Study 2a, participants were asked to enter a brand name. In our experimental conditions, participants were asked to either name a brand that fits positive communal traits (i.e., “family-oriented”, “trustworthy”, “friendly”, “sympathetic”, and “warm-hearted”) or negative communal traits (i.e., “dominant”, “egoistic”, “unkind”, “conceited”, and “stingy”). In Study 2b, we excluded “warm-hearted” to decrease the possibility that any temperature effects were due to semantic associations between “warmth” and our dependent variable of temperature association. In Study 2c we changed the priming words (for the positive communal condition: “sympathetic”, “helpful”, “trustworthy”, “friendly”, “compassionate”; negative: “dominant”, “egoistic”, “unkind”, “conceited”, “stingy”).

In all three studies, we then asked our participants to think about the brand as much as they could, and to describe their perceptions and feelings of the brand in at least 200 characters. They then proceeded to the same temperature estimation task as in Studies 1a&b. Again, at the end, participants rated whether they thought brands were fitting with the 20 personality traits from our first pretest on a 7-point scale (1 = *strongly disagree*, 7 = *strongly agree;* Study 2a Cronbach’s **α** COM_pos_ =. 96; COM_neg_ =. 91; AGE_pos_ =. 75; AGE_neg_ =. 75; Study 2b **α** COM_pos_ =. 80; COM_neg_ =. 64; AGE_pos_ =. 71; AGE_neg_ =. 76; Study 2c **α** COM_pos_ =. 97; COM_neg_ =. 92; AGE_pos_ =. 77; AGE_neg_ =. 79). For these studies, we used these scores to assess whether participants followed the instructions.

### Results

As we expected, in Study 2a temperature estimates were higher in the positive communal condition (*M* = 71.05, *SD* = 3.79) than the negative communal condition (*M* = 70.25, *SD* = 3.72), albeit marginally/nonsignificant, Cohen’s *d* = 0.21, *t*(301) = 1.84, *p* =. 066, and again no effect on subjective warmth, *t*(304) = -.34, *p* =. 74. Confirming Study 2a, but now without “warm-hearted” as priming word, Study 2b showed that temperature estimates were higher in the positive communal condition (*M* = 70.47, *SD* = 3.67) than in the negative communal condition (*M* = 69.82, *SD* = 3.82), Cohen’s *d* = 0.17, *t*(555) = 2.03, *p* =. 043, with again no effects on subjective warmth, *t*(555) =. 94, *p* =. 35. In Study 2c, we did not obtain the hypothesized effect (Cohen’s d = -.08, t(534.79) = -.89, p =. 38 (and again no effect on subjective warmth, t(542) = 1.6, p =. 11). It may be that these priming words could not elicit the hypothesized effect. However, given that we obtained the effect in four studies, statistically one should expect one failed study. Thus, the fact that one study failed makes the overall package more credible, not less (see e.g., [41]). On the basis of our Studies 1a and b, one may expect a mediation analysis for Studies a and b. Indeed, communality estimates may well mediate the relationship between communality condition and temperature estimates. This was however not the case. The reason why the communality estimates did not mediate between condition and temperature estimates should be sought in the status of the variable: In Studies 2a and b, we explicitly asked participants to come up with a positive versus negative communal brand, and the communality estimate is thus a manipulation check. In Studies 1a and b, the communality estimate is based on participants’ perception of the brand we have provided them. And, in fact, the difference in communality estimates was greater in Studies 2a (*d*’s CI95, 4.25–5.30) and 2b (*d*’s CI95 4.66, 5.35) than Study 1a (*d*’s CI95, 1.64–2.20). Moreover, regression analyses including communality estimates and condition revealed multicollinearity in Studies 2a (Tolerance =. 149) and 2b (Tolerance =. 138), but not in Study 1a (.510).

## Meta-Analysis

**Fig 1:**
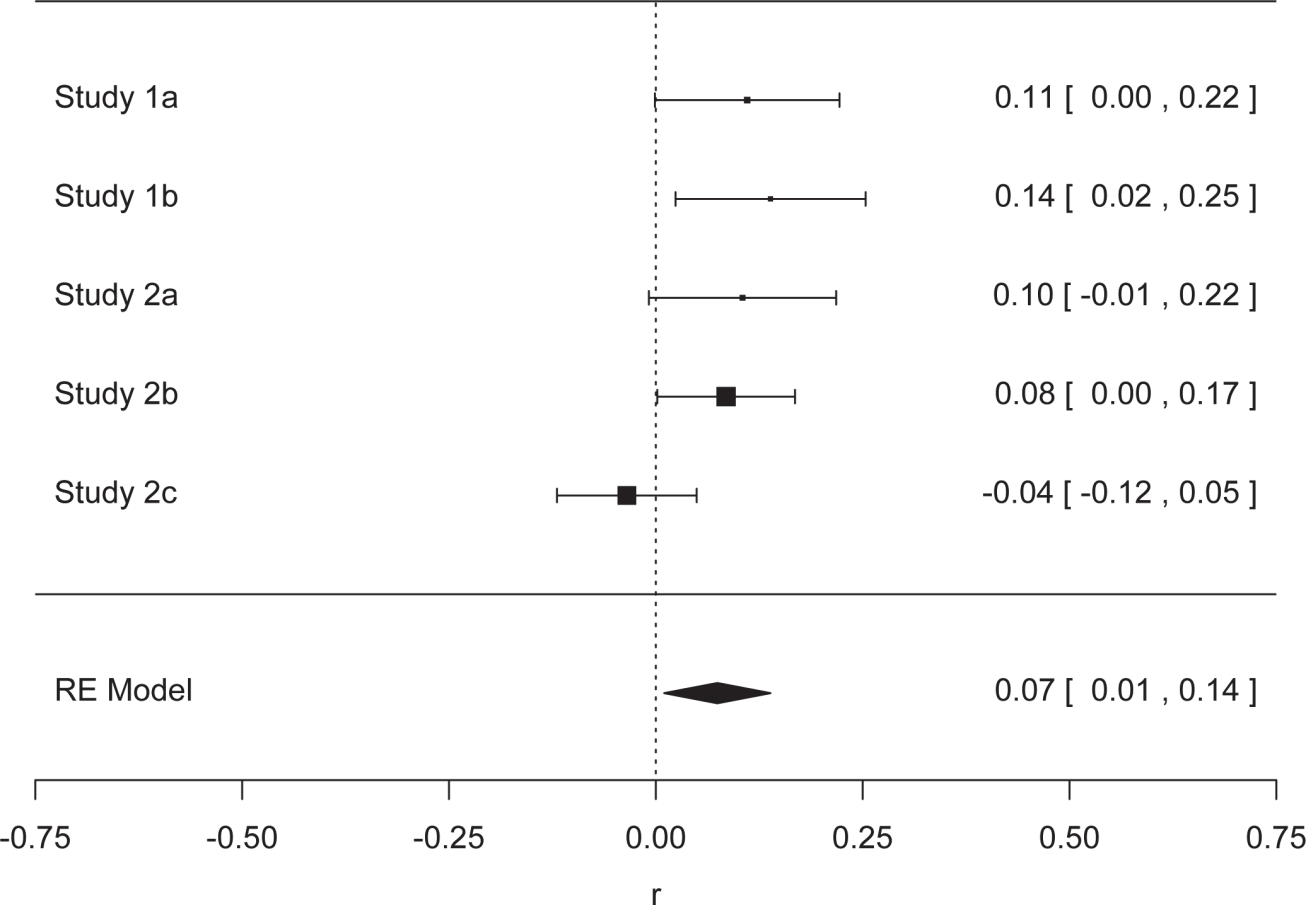
Forest plot of Studies 1a, 1b, 2a, 2b, and 2c of Temperature Estimates.

**Fig 2:**
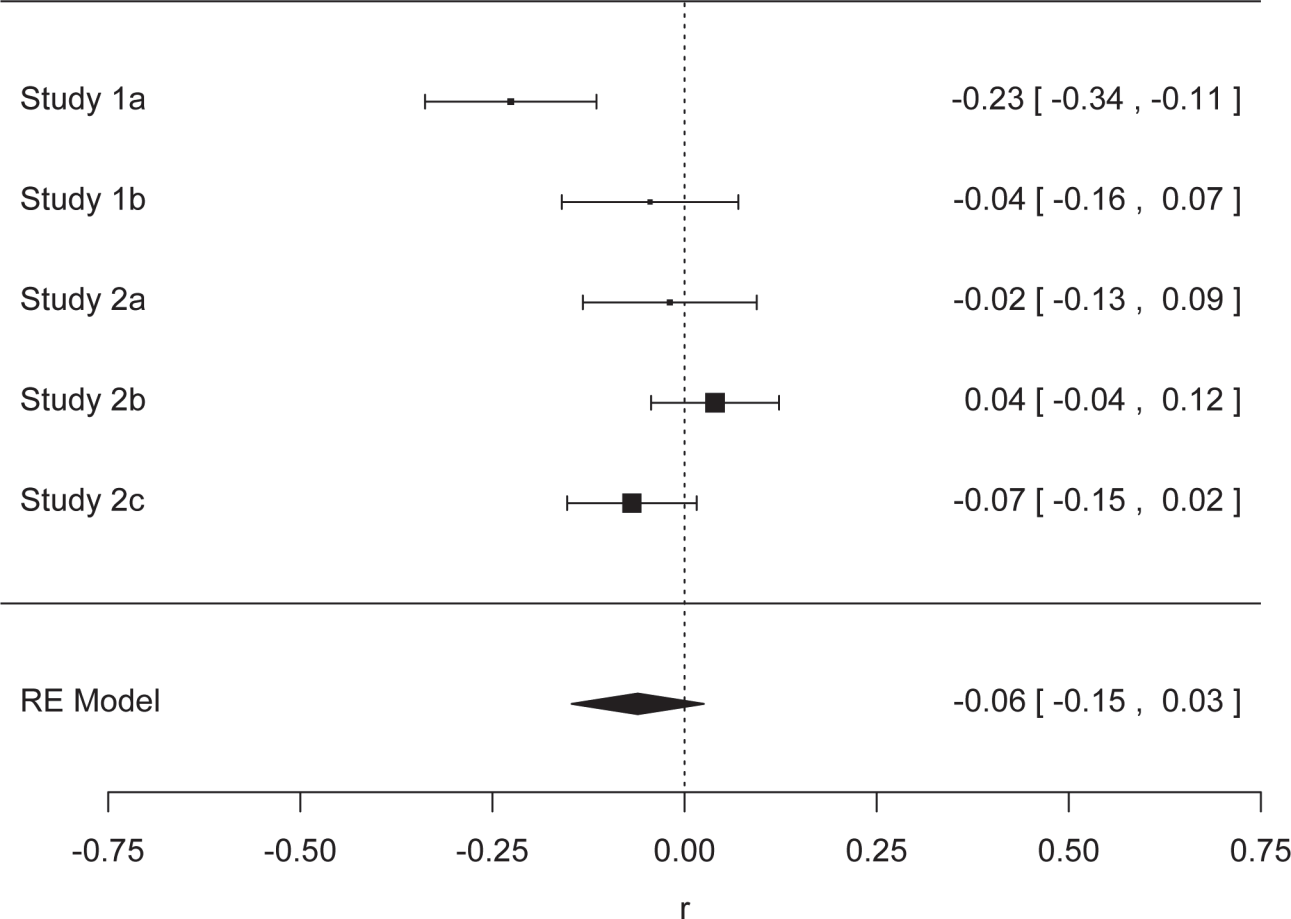
Forest plot of Studies 1a, 1b, 2a, 2b, and 2c of Subjective Warmth.

We conducted a meta-analysis with the metafor package in R [42] (see also [43]) to derive the overall mean effect size of averaged communality on temperature estimates. We conducted two meta-analyses, both with comparable results. Our first meta-analysis included the four studies reported in the main text. In order to obtain temperature estimates for Studies 1a and b that are comparable to the means of the positive and negative communal conditions in Studies 2a, 2b and 2c, we first calculated their averaged communality scores (*M*_*neg* =_ 2.3087; *M*_*pos*_ = 5.6812). We calculated the temperature estimates for those scores in Study 1a (High Communality = 69.20; Low Communality = 68.27; *SD* = 3.595) and Study 1b (High Communality = 71.36; Low Communality = 69.75; *SD* = 3.2839). The random effects meta-analysis produced a mean effect *r* =. 11, 95% CI,. 05,. 18. We also conducted similar meta-analyses for subjective warmth, and found that our confidence interval overlapped with 0 (*r* = -.06, 95% CI,-.17,. 05—Fig 1; *r* = -.06, 95% CI,-.15,. 03—Fig 2), meaning that there was no reliable effect related to subjective warmth in our sample.

## General Discussion

In five studies, we find that priming people with communal brands leads participants to estimate temperature as higher, with no consistent effect on subjective warmth. We found increased temperature perceptions by offering brands that were known to participants (Studies 1a&b), relating their perceived communality of the brand to temperature, and by letting participants choose a brand they thought to be positively or negatively communal (Studies 2a&b&c). The popularity of the Coca-Cola commercial may thus well be explained by its contrast with winter’s cold. This package of studies is important and timely, because of the doubts that have risen around comparable effects. Hopefully, the public availability of our methods and data will inspire solid close and conceptual replications [44, 45].

But there is one important question that still remains. That is, what exactly is the function of these effects? And, specifically, why was subjective warmth not influenced, as one may have expected? Elsewhere, we are suggesting that effects related to warmth and social relationships are rooted in people’s needs to thermoregulate themselves, with its roots in regulation of metabolic resources [23, 46]. The regulation of one’s body temperature is crucial for survival, and may well have extended to the regulation of close relationships more generally. This *may* also explains the differential effects for subjective warmth versus temperature estimations. The change in ambient temperature may well be because people’s internal models are activated that relate to thermoregulation, and the temperature estimate serves as a gage for the individual’s available resources, allowing the individual to exert predictive control over thermoregulation in which others (and thus also brands) can support. This temperature gage—unlike one’s internal sensations of temperature—thus provides a sort of weather report for the near “temperature future”. And we think that this gage serves for the regulation of one’s energy as postulated by Social Baseline Theory [46, 47]. Based on our meta-analysis, we can conclude that there is no reliable relationship between communality and subjective warmth. We conducted two analyses to explore this idea, with merged datasets 1a&b and 2a&b (so as to achieve sufficient power). For Studies 1a&b was, beyond a significant relationship between communality and temperature perception (*sr* =. 119, *t*(600) = 2.99, *p* =. 003, B =. 496) and a significant relationship between subjective warmth and temperature perception (*sr* =. 167, *t*(600) = 4.18, *p* <. 001, *B* =. 635), a significant interaction between subjective warmth and communality of the brand, *sr* =. 098, *t*(600) = 2.45, *p* =. 014, *B* =. 376. Simple slopes revealed that the relationship between communality and temperature estimates was there for people high on subjective warmth (*sr* =. 158, *t*(600) = 3.98, *p* <. 001, *B* =. 872), and absent for people low on subjective warmth (*sr* =. 021, *t*(600) =. 52, *p* =. 604, *B* =. 102). Surprisingly, the interaction effect was absent for our two priming studies (*sr* = -.007, *t*(856) = -.224, *p* =. 823). It may be that the priming of a communal brand permits a greater amount of future resources for all participants, whereas our correlational studies simply reflected a relationship of how much participants that are low on subjective warmth expect from their social world. This idea however needs further investigation.

Note that the divergence between such measurement is theoretically consistent with other work that is based on such *economy of action* principles, like the perception of slant [48, 49, 50]. Future research should thus also start investigating the role of fasting glucose levels and correlate these to subjective warmth measures and temperature estimates. Our theoretical ideas can then be extended to application: How can temperature estimates influence how people perceive products, how do these products regulate people’s metabolic resources, and to what degree does this vary for brands that sell better if the product is best served by being perceived communal versus agentic?

We do think there is a reason why thermoregulation is implicated in social relationships so frequently. We think it is likely that the effects that we have found here are reliant on evolutionarily prepared mechanisms and expressed through alterations in the autonomic nervous system, as social exclusion leads to skin temperature drops [51]. This is important, as the association between warmth and social relations is likely ubiquitous. Nevertheless, although it is likely that humans are predisposed to seek warm experiences, learning occurs throughout one’s lifetime through the alteration of one’s internal “temperature working models”, as the relationship between warmth and cognition and behavior have been found to be moderated by individual relationship experiences, both amongst young children [52] and in adulthood [53].

There are still a number of caveats to the present research. First, the effect size across studies is small (*r* =. 13). We do not think that this is surprising, and the first reason for it being so is that we conducted our studies with very low experimental control. In fact, it may perhaps be even more surprising that we find these effects *at all*. Nevertheless, small effects are still relevant for consumers’ relationships with brands if very subtle and cheap manipulations could lead to increased sales, and to better regulation of resources through consumer products. The second caveat could be that in our nonsignficant study we changed our priming words. However, our meta-analysis in- versus excluding this study changes little to the overall effect of the meta-analysis. Although it is possible that the nonsignificant effect was due to changing the priming words, it seems more likely the nonsignificant effect was a statistical fluke, making the overall effect more credible [43].

In answering the relevance of social thermoregulation to relationships with brands, we should investigate the relationship between communality, temperature perceptions, and how likely they are to be willing purchase from the chosen brands. We explored this issue in Studies 1a and b. We asked participants to what degree they were likely to purchase from this particular brand. In explorative analyses, we found two significant mediations. First, using a bootstrap analysis with 1000 resamples [54] revealed a positive relationship between communality and willingness to purchase (*b* = 16.05, *SE* = 1.11, *t* = 14.49, *p* <. 01), which slightly weakened when inserting temperature estimates (*b* = 15.81, *SE* = 1.11, *t* = 14.21, *p* <. 01), with both the path from communality to temperature estimates (*b* =. 45, *SE* =. 17, *t* = 2.72, *p* <. 01) and temperature estimates to willingness to purchase (*b* =. 54, *SE* =. 27, *t* = 2.01, *p* =. 05) significant. Consistent with a mediation effect, the CI did not overlap with 0 (indirect effect =. 03, 95% CI,. 03,. 67). We obtained a comparable effect for how close the brand was to people’s identities (for analyses, see OSF, 2014). Thus, our exploratory analyses suggest that to what degree a brand makes people feel literally warm really matters in people’s purchasing decisions.

A further direction for future research concerns distinguishing between people’s concepts of relationships with brands and people—and this is the second explanation for our small effect size. Furthermore, it should be kept in mind when people explore how brands can regulate people’s emotions. Indeed, researchers have found that close others can regulate people’s emotions through a warm touch [46]. Could brands be as fulfilling as people’s relationship with other human beings? We think this is rather unlikely for two reasons: First, MRI studies suggest differential patterns of brain activation for human versus brand descriptor judgments [55]. Second, we expect that the effect size for comparable studies on close others will be (much) larger. Future research should address brands’ potential for the regulation of the self, and whether we are wrong about it being able to imbue a relationship with the same meaning as other human beings can.

Although there have been raising concerns about the stability of comparable findings [56; 25], the present studies quite strongly support the relationship between temperature estimates and social relations. We therefore confidently recommend these studies for further empirical research and for practitioners who are interested in marketing research. As a closing statement, we would like to emphasize that, much similar to human relationships, while letting *appear* a brand as psychologically/physically warm seems attractive on the short run, it seems that “sincerely warm” brands over time will benefit not only the producer most, but also the consumer [57].
